# Weed-Hoeing Periods in Cowpea Cultivation under Direct and Conventional Systems

**DOI:** 10.3390/plants12142668

**Published:** 2023-07-17

**Authors:** Eudinete Ribeiro de Sousa, Larissa de Oliveira Fontes, José Hamilton da Costa Filho, Antonio Leandro Chaves Gurgel, Wéverson Lima Fonseca, Alan Mario Zuffo, Daniele Galvão Alencar, Tairon Pannunzio Dias e Silva, Julian Junio de Jesús Lacerda, Jorge González Aguilera, José Antonio Rodríguez García, Renatto Nicolino Motta Zevallos, Luis Morales-Aranibar, Alfredo Modesto Marcavillaca Luna, Hebert Hernán Soto Gonzales

**Affiliations:** 1 Department of Agronomy, Universidade Federal do Piauí, Bom Jesus 64915-000, PI, Brazil; eudinethapce@hotmail.com (E.R.d.S.); weversonufpi@gmail.com (W.L.F.); danielegalvao.flp@gmail.com (D.G.A.); julian@ufpi.edu.br (J.J.d.J.L.); 2 Department of Agronomy, Universidade Federal do Rio Grande do Norte, Macaíba 59280-000, RN, Brazil; hamilton_costa@yahoo.com.br; 3 Department of Zootechnics, Universidade Federal do Piauí, Bom Jesus 64915-000, PI, Brazil; antonio.gurgel@ufpi.edu.br (A.L.C.G.); pannunzio@ufpi.edu.br (T.P.D.e.S.); 4 Department of Agronomy, State University of Maranhão, Balsas 65800-000, MA, Brazil; alanzuffo@professor.uema.br; 5 Department of Crop Science, State University of Mato Grosso do Sul, Cassilândia 79540-000, MS, Brazil; j51173@yahoo.com; 6 Escuela Profesional de Ingeniería Ambiental, Universidad Nacional de Moquegua (UNAM), Ilo 18601, Peru; jrodriguezg@unam.edu.pe (J.A.R.G.); hsotog@unam.edu.pe (H.H.S.G.); 7 National Intercultural University of Quillabamba, La Convenciòn, Cusco 084-282728, Peru; renatto.motta@uniq.edu.pe (R.N.M.Z.); luis.morales@uniq.edu.pe (L.M.-A.); alfredo.marcavillaca@uniq.edu.pe (A.M.M.L.)

**Keywords:** *Vigna unguiculata*, mechanical control, interference, competition, yield

## Abstract

Weed coexistence with an agricultural crop can negatively affect its growth, development, and yield. From this perspective, this study aimed to evaluate weed management strategies and their effect on the agronomic parameters of cowpea cultivation in direct (SPD) and conventional (SPC) planting systems. The experiment was set up in a completely randomized block design with a split-plot arrangement with four replications. The plots received a source of variation referring to the planting systems (direct and conventional planting), and the subplots corresponded to ten weed management strategies (manual hoeing 18 days after planting (DAP); at 36 DAP; at 54 DAP; at 18 and 36 DAP; at 18 and 54 DAP; at 18 and 72 DAP; at 36 and 54 DAP; at 36–72 DAP; at 18, 36, and 54 DAP; and a control with no hoeing). Density and dry mass evaluations of the cowpea plants were performed at harvest (72 DAP) by determining the number of pods per plant, pod length, number of grains per pod, 1000-grain mass, and yield. A total of 28 species distributed in 12 botanical families were identified in the two cultivation systems. The family *Poaceae* showed the highest frequency, with 25% of the species identified. At the end of the assay, treatment 20 had the highest positive influence and provided significant quantitative gains to the complex of traits related to cowpea production (SPD and hoeing at 18, 36, and 54 DAP). UPGMA cluster analysis and canonical discriminant analysis were performed and allowed a better classification of the evaluated treatments. It was observed that the first two canonical variables explained 90.8% of the total variance contained in the original variables. The use of SPD with weeding at 18, 36, and 54 days after planting provides greater weed control and significant quantitative gains for the complex of characteristics related to cowpea production. The results underscore the importance of choosing the correct cropping system and implementing effective weeding practices to optimize weed control and improve crop performance.

## 1. Introduction

Cowpea (*Vigna unguiculata* (L.) Walp.), a crop widely consumed in African countries, has garnered the interest of breeders on a global scale in recent times [1]. In Brazil, commonly known as “cowpea macassar”, “cowpea-de-corda”, or “cowpea-caupi”, it is a legume species with a high protein content and expressive socioeconomic importance as one of the most consumed grains in Brazil, where it is widely grown in the north and northeast regions of the country due to its adaptation to local edaphoclimatic conditions [2]. It is widely grown in the northern and northeastern regions of the country due to its adaptation to local edaphoclimatic conditions, where resistance to abiotic stress conditions stands out, such as the water deficit characteristic of semiarid regions [3].

The area cultivated with cowpea “caupi” in the 2023 harvest in Brazil was 381 thousand ha, and the average productivity was 396 kg ha^−1^ [4]. Among the main factors that affect cowpea yield, the interference imposed by weeds stands out due to competition for water, light, and resources, resulting in a lower quality of the product harvested and/or an increase in crop production costs, which is potentiated by the inexistence of commercial formulations for this crop registered with the Ministry of Agriculture, Livestock, and Supply of Brazil as of 2016 [5], thus preventing chemical weed control.

Yield reductions resulting from weed infestation in cowpea cultivation can reach up to 76%, with variations dependent on the specific cowpea cultivar, prevailing environmental conditions, and weed control techniques employed [6]. Furthermore, the negative interference of factors such as competition and allelopathy reduces the number of pods per plant, seeds per pod, and grain mass in addition to increasing operational costs with grain harvest, drying, and processing [7].

Manual hoeing in traditional farming regions is still one of the strategies adopted for weed control, showing high efficacy when weeds are still in an early growth stage and when the environmental conditions favor water loss by recently cut weeds [8]. However, the scarce manpower for performing agricultural management services and the onerous nature of this technique from an economic point of view highlight the need for studying and providing weed management alternatives for cowpea cultivation. De Campos et al. [9] demonstrated that the coexistence of cowpea with weeds results in up to 90% productivity losses, and the critical period of weed control in the semiarid region of Minas Gerais is from 11 to 36 days after crop emergence.

The direct plantation (SDP) method is a management strategy that allows better soil management with the possibility of performing minimal activities in the soil and the incorporation of mulch, promoting a better productive response of the plants [10,11]. Combined with productivity and soil improvement, it is a viable option to control weed communities, which changes the dynamics of weed infestation and leads to its reduction [10]. By adopting SDP, the layer of straw over the soil acts as a protective covering, reducing the exposure of the soil to sunlight, regulating its temperature, and decreasing the germination of weed seeds [11,12]. In addition, straw also acts as a physical barrier, affecting weed emergence or the release of substances that inhibit the germination and/or growth of weeds [13,14,15,16].

In this scenario, given the importance of cowpea and the need to optimize its yield to favor socioeconomic development and improve the quality of life of farmers in the southern region of the Brazilian State of Piauí (PI), the use of a cultivation system combined with manual weeding times can be a viable alternative in the control of weeds that compete with cowpea plants. This study aimed to evaluate the production of cowpea subjected to weed control using strategic periods for manual hoeing in stands cultivated under direct and conventional planting in the Vale do Gurguéia, Bom Jesus, PI.

## 2. Results and Discussion

The weed community of the studied area showed high diversity. A total of 28 species distributed in 12 botanical families were identified in the two cultivation systems.

The family *Poaceae* showed the highest frequency, with 25% of the species identified, followed by *Malvaceae*, with four species (14.9%); *Amaranthaceae* and *Euphorbiaceae*, with three species (10.72%); *Asteraceae*, *Rubiaceae*, and *Fabaceae*, with two species (7.14%); and *Covolvulaceae*, *Cucurbitaceae*, *Cyperaceae*, *Nyctaginaceae*, and *Portulacaceae*, with one species each (3.57%) (Table 1).

There was a higher predominance of eudicots, accounting for 71% of the species identified and represented by ten families encompassing twenty species. For the monocots, only two families were identified (*Cyperaceae* and *Poaceae*), encompassing eight species.

In a study carried out by Campos et al. [9] on the occurrence of weed species in the cowpea crop (cultivar BRS Itaim) in the semiarid region of Minas Gerais, Brazil, more than 1300 specimens of weeds, which were distributed in seven families and ten species, were found during the crop cycle. The weed community was composed of 70% dicots and 30% monocots. Considering the dicotyledonous species, the *Fabaceae* family stood out for having two species. Considering the monocotyledonous species, the *Poaceae* family stood out for having three species. The other families had only one species each.

The diversity of eudicot weeds was also studied by Cunha et al. [17] in an experiment on the phytosociology of infested bell pepper plants in direct (SPD) and conventional (SPC) planting systems. Variations in weed populations may occur due to edaphoclimatic differences, type of production system, previous crops, and weed seed banks in each region [18,19].

In the present study, the occurrence of the weed species *Digitaria sanguinalis*, *Eleusine indica*, *Panicum maximum*, *Phyllanthus niruri*, *Phyllanthus niruri*, *Mimosa pudica*, *Cyperus rotundus*, and *Portulaca oleracea* was observed only in the conventional planting system. These results demonstrate the importance of maintaining vegetation cover in reducing weed diversity.

The weed diversity and total dry mass accumulation were low in the SPD due to the smaller weed population in the area, corroborating the observations of Pacheco et al. [20] (Table 2).

In a study carried out by Sousa et al. [21] on a phytosociological survey of the weed community in cowpea cultivated in no-tillage and conventional tillage systems in the region of Vale do Gurguéia, municipality of Bom Jesus, PI, 27 species with a density of 65.24 plants m^−2^ were verified in the SPC. The highest frequencies were observed for *Alternanthera tenella*, *Mimosa pudica*, *Richardia brasiliensis*, *Bidens pilosa*, and *Cenchrus echinatus*, obtaining densities of 11.17, 4.69, 4.69, 4.61, and 4.22 plants m^−2^, respectively. In the cultivated area under NTS, 12 species were verified as distributed in 5 families, with a total density of 31.32 plants m^−2^. The species that stood out in terms of density in this planting system were *R. brasiliensis*, *A. tenella*, and *B. verticillata*, with 13.20, 3.83, and 2.89 plants m^−2,^ respectively. The species *R. brasiliensis* showed the highest relative frequency, occurring in 68% of the sampled areas.

The positive contrast observed in the SPD in relation to SPC suggests that mulching contributes to weed control. Vegetation cover on the soil surface acts to control weeds through physical effects, reducing and modifying the quality of light needed to stimulate germination [15] as well as representing a physical barrier capable of causing exhaustion in seedling reserve material during the initial development process [13,14,22]. Furthermore, plant residues can release allelopathic substances with a positive effect on the inhibition of weed germination and/or growth [15,16]. These effects may have occurred in this study, resulting in reduced competition by weeds with cowpea plants.

The effect of competition on cowpea yield also have negatively impacted the number of plants per pod in all treatments in the SPC system regardless of the hoeing intervals. The number of pods per plant is one of the most important components of cowpea yield since it is the most correlated variable with grain yield [23].

Matos et al. [24] also observed that the number of pods per plant was influenced by weed coexistence periods, which was reduced after 10 DAP, and by control strategies with positive results in treatments in which the crop was kept clean until 30 days after planting (DAP).

The results obtained by Oliveira et al. [25] corroborate the observations of this study, as they attest to a reduction in the number of pods for three cowpea cultivars with the increase in the weed coexistence period.

A possible explanation for the reduction in the number of pods refers to the competition between weeds and cowpea plants, leading to the lower emission of inflorescences or flower abortion [26].

When observing the relevant number of components related to production and the known importance of each of them to decision making about the best cultivation system associated with the ideal period for hoeing, the analysis of the multivariate characteristics associated with production becomes indispensable. According to the MANOVA, a significant effect was observed for the multivariate characteristic (*p* < 0.01), highlighting the existence of significant differences between treatments (Table 3).

Next, the generalized distance of Mahalanobis was estimated for the 20 treatments. The Euclidean distance, mean Euclidean distance, and the Mahalanobis distance are parameters often used in scientific studies on genetic improvement, and the results measure the genetic distance of the cultivars. However, this technique has been gradually used in other fields.

The Mahalanobis distance (D2) is very useful because it is analogous to other multivariate techniques [27] and has good efficiency in estimating distances between the evaluated treatments, allowing a good quantitative visualization of similarities between treatments and groups of treatments.

The highest distances were observed between treatment 20 (SPD and hoeing at 18, 36, and 54 DAP) and the others, resulting in the formation of similar treatment groups between each other and divergence between intergroups (Table 4).

**Figure 1:**
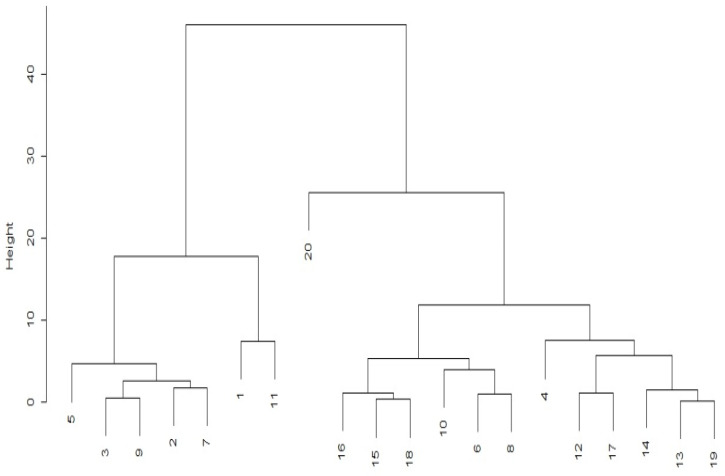
Divergent groups obtained based on the matrix of Mahalanobis generalized distances (D20 × 20) for the 20 treatments evaluated. Source: Authors (2023).

The UPGMA clustering analysis was processed based on the generalized distances of Mahalanobis, resulting in the formation of three groups with 90% similarity and contrasting with each other (Figure 1).

Group one (G1) was formed by treatments 5 (SPC and hoeing 18 at 36 DAP), 3 (SPC and hoeing at 36 DAP), 9 (SPC and hoeing at 36 and 72 DAP), 2 (SPC and hoeing at 18 DAP), and 7 (SPC and hoeing at 18 and 72 DAP).

Group two (G2) was formed by treatments 16 (SPD and hoeing at 18 and 54 DAP), 15 (SPD and hoeing at 18 and 36 DAP), 18 (SPD and hoeing at 36 and 54 DAP), 10 (SPC and hoeing at 18, 36, and 54 DAP), 6 (SPC and hoeing at 18 and 54 DAP), and 8 (SPC and hoeing at 36 at 54 DAP).

Group three (G3) was formed by treatments 4 (SPC and hoeing at 54 DAP), 12 (SPD and hoeing at 18 DAP), 17 (SPD and hoeing at 18 and 72 DAP), 14 (SPD and hoeing at 54 DAP), 13 (SPD and hoeing at 36 DAP), and 19 (SPD and hoeing at 36 and 72 DAP).

Group four (G4) was formed by treatments 1 (SPC and no hoeing) and 11 (SPD and no hoeing).

Group five (G5) was formed only by treatment 20 (SPD and hoeing at 18, 36, and 54 DAP), directly contrasting with G1 and G2. The shortest distance was observed between treatment 20 and a treatment belonging to another group (treatment 15), which used SPD but with hoeing until 36 DAP.

A high magnitude of the intergroup distance was also observed between treatments 10 and 20 (9.15), with SPC and hoeing at 18, 36, and 54 DAP and SPD and hoeing at 18, 36, and 54 DAP, respectively, evidencing the influence of the planting system on the contrasts observed between treatments even when using the same hoeing intervention periods. This indicates that the no-tillage method had a significant influence on the observed contrasts between these treatments, even when the same weeding interventions were performed.

The highest distance observed occurred between treatments 20 and 1, corresponding to SPD and hoeing at 18, 36, and 54 DAP and no hoeing, respectively, highlighting the significant influence of the cultivation systems and hoeing interventions on this parameter. The distance between these treatments suggests that the cultivation system and weeding have a considerable impact on the traits evaluated in the study.

Group G4 included the control treatments to evaluate the effect of hoeing interventions performed throughout the crop cycle. The high magnitudes of the distances observed between treatments 1 and 11 in relation to the others highlight the need for hoeing for weed control regardless of the cultivation system.

**Figure 2:**
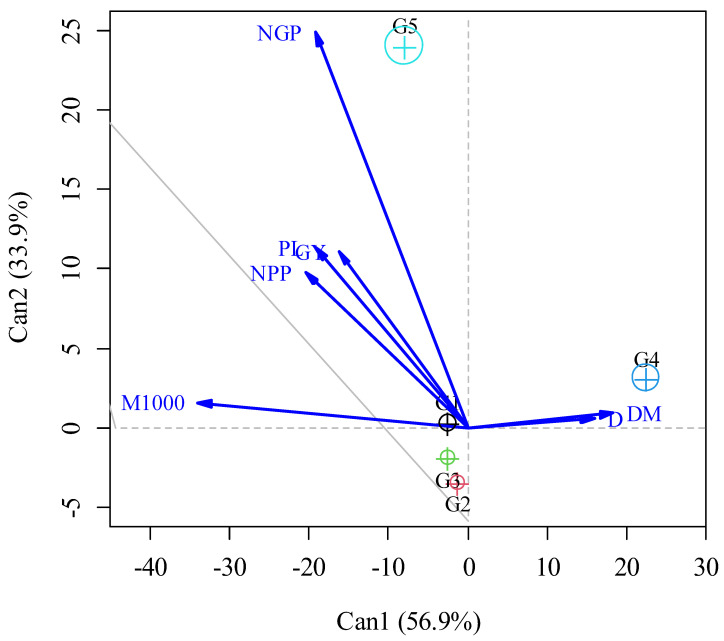
Graph depicting the canonical discriminant analysis (canonical variables Can1 and Can2) for the weeds variables, i.e., density (D) and dry mass (DM), and cowpea variables, i.e., number of pods per plant (NPP), number of grains per pod (NGP), pod length (PL), thousand-grain mass (M1000), and grain yield (GY). The round symbols with a sign but in the center and different colors are related to the groups formed, in black to G1, in red to G2, in green to G3, in blue to G4, and in magenta green to G5.

By canonical discriminant analysis, it was observed that the first two canonical variables explained 90.8% of the total variance contained in the original variables (Figure 2). Among the evaluated treatments, the G5 group, formed only by treatment 20 (SPD and hoeing at 18, 36, and 54 DAP), presented the highest means for the variables (NPP, NGP, PL, M1000, and GY) of cowpea in addition to providing the greatest reductions in the variables (D and DM) of weeds. In the treatments of the G4 group, formed by treatments 1 (SPC and no hoeing) and 11 (SPD and no hoeing), there were the highest values of D and DM of weeds and, at the same time, lower averages of productive development of cowpea plants. Overall, these observations suggest that the planting system, weeding interventions, and no weeding had a significant impact on the traits evaluated in the study. The results underscore the importance of choosing the right cropping system and implementing effective weeding practices to optimize weed control and improve crop performance.

## 3. Materials and Methods

### 3.1. Location

The experiment was conducted in the municipality of Bom Jesus, PI, located at 09°04′28″ S and 44°21′31″ W, at an elevation of 277 m a.s.l. The municipality is part of the semiarid region of Piauí, showing a hot and humid climate classified by Köppen as Cwa (temperate with dry winters and summer and autumn rainfall).

The experiment was set up in a completely randomized design with a split-plot arrangement with four replications. The plots consisted of a variation source referring to the planting system (direct and conventional planting), and the subplots received ten weed management strategies (manual hoeing 18 days after planting (DAP); at 36 DAP; at 54 DAP; at 18 and 36 DAP; at 18 and 54 DAP; at 18 and 72 DAP; at 36 and 54 DAP; at 36–72 DAP; at 18, 36, and 54 DAP; and a control without hoeing).

### 3.2. Experimental Material and Implementation of the Experiment

Straw formation for direct planting occurred in August 2017 using 1 kg ha^−1^ of seeds of a corn variety employed in traditional agriculture in the municipality of Bom Jesus, PI.

The seeds were distributed with single superphosphate (5 kg ha^−1^) and the NPK combination 54–30–40 (40 kg ha^−1^) at the bottom of the planting holes. The experimental units consisted of six rows 3.0 m long, spaced 0.50 m apart, with plants spaced 0.50 m apart in the rows. The four central rows formed the useful plot, except for the 0.50 m stretch at the ends of each row.

After the corn harvest in November 2017, the crop remains were distributed around the base of the corn plants to form the mulch. At the time of cowpea sowing, the mulch composed of corn remains was quantified based on subsamples taken using a hollow square-shaped template with sides measuring 0.4 m. Next, the samples were dried to constant weight in a forced-air oven at 65°C, thus determining the amount of 2.0 t ha^−1^ of dry matter used in the assay.

In the area corresponding to conventional planting, soil preparation was performed by plowing and double harrowing, performed a week before cowpea sowing.

The soil of the experimental area was classified as a Dystrophic Yellow Ferrasol based on a soil analysis performed with a composite sample collected at the 0 to 20 cm depth layer. The chemical analysis (Table 5) was performed at the Laboratory of Soils of the Federal University of Piauí.

Next, liming was performed using 450 g of dolomitic limestone per plot 30 days before sowing. After this period, supplementary fertilization was performed according to the chemical soil analysis, following the technical recommendations for cowpea [28] (Table 6).

The experiment with cowpea was established in late November 2017 through direct sowing of the cultivar BRS Imponente.

Before planting, the seeds were treated with an insecticide composed of an active ingredient and a fungicide composed of an active ingredient based on carbendazim and tiram. One seed was planted per hole at a depth of 5 cm, and a sprinkler irrigation system was adopted, with a watering schedule of two equidistant irrigation events per week and suppression on rainy days.

**Figure 3:**
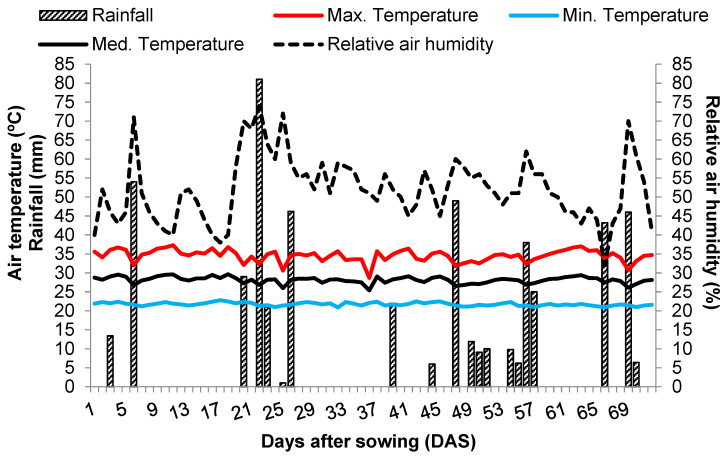
Meteorological data of the experimental area during the implementation of the experiment in Bom Jesus, PI, UFPI, 2018. Source: Authors (2023).

The local meteorological characterization during the experiment was used to guide the necessary management and crop practices, e.g., chemical control of pests and diseases (Figure 3).

### 3.3. Variables Determined

At the time of cowpea harvest, the plant density (plants per m^2^) and dry matter (g ha^−1^) of weeds were evaluated.

For each cultivation system, 60 samples were collected using the 0.16 m² square-shaped template in the useful area of each plot. Every time this procedure occurred, the weeds were cut at ground level, sampled, separated by species, counted, and dried to constant weight in a forced-air oven at 65 °C.

The cowpea pods, collected from the useful area of the plots, were harvested daily using the beginning of maturity as a criterion, i.e., the moment when the pods showed an intense yellow color. After harvesting, the pods were used to determine the number of pods per plant (NPP), pod length (PL) in centimeters (cm), number of grins per pod (NGP), thousand-grain mass (M1000) in grams (g), and grain yield (GY).

The number of pods per plant was obtained by summing the pods harvested during the crop cycle and dividing this total by the number of plants in the useful area. The yield was estimated as the ratio of the grain mass harvested (kg) to the total harvested area (m²). Then, the yield data were converted into kg ha^−1^.

### 3.4. Statistical Analysis

Once the assumptions of homoscedasticity, normality, additivity, and independence were verified, the data obtained were subjected to analysis of variance through the F test (*p* < 0.05) using the software R version 3.0.1 [29] and the ExpDes package [8].

For the multivariate characteristics, multivariate analysis of variance (MANOVA) was processed with the Pillai method, which is the most robust method for samples or groups with small and different dimensions [30]. To verify the divergence between the twenty treatments obtained through all possible combinations between the ten hoeing periods and two cultivation systems, a generalized distance matrix of Mahalanobis (D20 × 20) was used to process the UPGMA clustering analysis. Both statistical procedures were performed using the software R version 3.0.1 [29].

To discriminate the treatment groups as a function of the variables, canonical discriminant analysis was performed, represented by a biplot graph constructed for the first two canonical variables. Furthermore, ellipses with 95% confidence were constructed to detect significant differences (*p* < 0.05) between treatment groups. All analyses were performed with R software version 3.6.1 [29]. Canonical discriminant analysis was performed using the candisc package [31].

## 4. Conclusions

The use of direct cultivation with weeding at 18, 36, and 54 days after planting provides a greater positive influence on weed control and significant quantitative gains for the complex of characteristics related to cowpea production.

## Figures and Tables

**Table 1:** Weed distribution per family and species in two cowpea cultivation systems (direct planting and conventional cultivation). Bom Jesus, PI, UFPI, 2018.

Family (%)	Scientific Name	Brazilian Common Name	Class	Planting System
Poaceae (25.00)	*Andropogon gayanus*	Capim andropogon	M	CD
	*Axonopus purpusii*	Capim mimoso	M	CD
	*Brachiaria plantaginea*	Capim marmelada	M	CD
	*Cenchrus echinatus*	Capim carrapicho	M	D
	*Digitaria sanguinalis*	Capim colchão	M	C
	*Eleusine indica*	Capim pé de galinha	M	C
	*Panicum maximum*	Capim colonião	M	C
Malvaceae (14.29)	*Sida cordifolia*	Malva branca	E	CD
	*Sida glaziovii*	Malva guanxuma	E	CD
	*Sida rhombifolia*	Malva relógio	E	CD
	*Waltheria americana*	Malva veludo	E	CD
Amaranthaceae (10.72)	*Alternanthera tenella*	Apaga-fogo	E	CD
	*Amaranthus deflexus*	Caruru-rasteiro	E	CD
	*Amaranthus viridis*	Caruru de mancha	E	CD
Euphorbiaceae (10.72)	*Euphorbia hirta*	Erva de Santa Luzia	E	CD
	*Cnidosculus pubescens*	Cansanção	E	CD
	*Phyllanthus niruri*	Quebra pedra	E	C
Asteraceae (7.14)	*Bidens pilosa*	Picão preto	E	CD
	*Conyza bonariensis*	Buva	E	CD
Rubiaceae (7.14)	*Borreria verticillata*	Vassourinha de botão	E	CD
	*Richardia brasiliensis*	Poaia branca	E	CD
Fabaceae (7.14)	*Anadenanthera colubrina*	Angico branco	E	CD
	*Mimosa pudica*	Malícia	E	C
Convolvulaceae (3.57)	*Ipomoea* sp.	Corda de viola	E	CD
Cucurbitaceae (3.57)	*Momordica macrophylla*	Melão de são Caetano	E	CD
Cyperaceae (3.57)	*Cyperus rotundus*	Tiririca	M	C
Nyctaginaceae (3.57)	*Boerhavia diffusa*	Pega pinto	E	CD
Portulacaceae (3.57)	*Portulaca oleracea*	Beldroega	E	C

CD, conventional and direct; C, conventional; D, direct; E, eudicot; M, monocot. Source: Authors (2023).

**Table 2:** Individual means for the density (D) and dry mass (DM) of weeds, number of pods per plant (NPP), number of grains per pod (NGP), pod length (PL), thousand-grain mass (M1000), and grain yield (GY) of cowpea for the 20 treatments evaluated.

Number ^1^ Treatments			D	DM	NPP	NGP	PL	M1000	GY
1	Conventional planting	No hoeing	76.29	1874.84	1.39	6.25	15.35	10.51	53.20
2		Hoeing at 18 DAP	65.31	1509.22	5.25	8.75	16.70	196.43	332.93
3		Hoeing at 36 DAP	51.88	1122.50	6.20	9.38	16.98	198.64	419.71
4		Hoeing at 54 DAP	36.45	416.56	4.88	7.75	16.20	175.42	224.51
5		Hoeing at 18 and 36 DAP	59.81	1146.72	12.36	10.33	18.10	233.52	1264.80
6		Hoeing at 18 and 54 DAP	38.89	586.09	11.64	9.60	17.85	220.36	897.26
7		Hoeing at 18 and 72 DAP	64.70	1542.50	11.53	9.53	17.60	204.78	589.71
8		Hoeing at 36 and 54 DAP	35.83	802.03	12.09	9.83	17.90	221.54	1195.73
9		Hoeing at 36 and 72 DAP	52.29	1099.38	9.22	9.48	17.20	202.56	429.17
10		Hoeing at 18, 36, and 54 DAP	32.52	663.89	19.45	11.05	19.20	265.30	1751.28
11	Direct planting	No hoeing	47.78	1166.88	4.20	7.98	16.45	23.77	144.96
12		Hoeing at 18 DAP	38.45	916.09	11.23	8.95	16.90	208.07	424.53
13		Hoeing at 36 DAP	25.63	768.28	11.73	9.90	17.00	210.26	532.93
14		Hoeing at 54 DAP	18.14	315.16	8.46	8.90	16.60	207.66	308.60
15		Hoeing at 18 and 36 DAP	24.26	642.76	18.50	11.15	18.90	235.35	1433.28
16		Hoeing at 18 and 54 DAP	19.14	378.44	16.95	10.30	18.30	227.27	1056.62
17		Hoeing at 18 and 72 DAP	39.72	1079.06	15.92	10.15	17.30	213.79	634.32
18		Hoeing at 36 and 54 DAP	18.56	433.44	17.03	10.70	18.79	231.86	1365.18
19		Hoeing at 36 and 72 DAP	23.80	670.31	12.89	10.10	17.40	211.58	571.86
20		Hoeing at 18, 36, and 54 DAP	19.53	394.06	24.98	18.40	20.20	291.68	2066.04

^1^ number and identification of the treatment; DAP, days after planting. Source: Authors (2023).

**Table 3:** Summary of the MANOVA for the multivariate characteristics associated with cowpea production features: density (D) and dry mass (DM) of weeds, number of pods per plant (NPP), number of grains per pod (NGP), pod length (PL), thousand-grain mass (M1000), and grain yield (GY).

Source of Variation	^1^ GL	^2^ Pillai	^3^ Aprox. F	^4^ GLN	^5^ GLD
Intercept	1	0.992 **	1216.920 **	7	71
Treatments	1	0.689 **	22.520 **	7	71
Blocks	1	0.045	0.48	7	71
Residual	77				

^1^ Degrees of freedom; ^2^ Pillai test; ^3^ F test; ^4^ degrees of freedom of the numerator; ^5^ degrees of freedom of the denominator. ** Significant by the F test of Shapiro—Wilk at 1% probability. Source: Authors (2023).

**Table 4:** Matrix of Mahalanobis generalized distances (D20 × 20) between the 20 treatments evaluated.

	1	2	3	4	5	6	7	8	9	10	11	12	13	14	15	16	17	18	19	20
1	-																			
2	11.17	-																		
3	11.98	1.58	-																	
4	16.86	9.16	3.70	-																
5	12.33	4.45	4.12	10.32	-															
6	16.46	8.31	3.20	2.38	4.86	-														
7	10.72	1.78	2.22	9.58	3.46	6.13	-													
8	16.96	10.39	5.11	5.21	4.63	1.01	7.75	-												
9	12.53	2.58	0.50	3.76	4.66	2.63	1.55	4.92	-											
10	25.14	18.92	11.78	11.72	7.78	3.95	12.53	1.94	10.38	-										
11	7.42	17.84	12.18	9.71	16.56	9.93	14.56	10.45	11.24	15.98	-									
12	18.24	8.48	3.47	3.13	9.48	2.22	5.59	4.11	1.96	8.06	10.42	-								
13	25.01	15.53	7.88	5.15	15.14	4.10	11.58	5.12	6.08	8.03	11.44	1.37	-							
14	29.80	19.00	10.00	3.71	19.59	5.43	16.76	7.61	8.73	11.92	13.82	3.30	1.44	-						
15	27.73	22.34	13.68	11.18	13.60	4.94	15.48	3.38	11.48	1.71	13.15	6.46	4.27	7.58	-					
16	30.74	24.09	14.21	8.85	17.77	5.25	17.64	5.29	11.62	4.71	13.22	5.16	2.38	3.79	1.18	-				
17	20.64	12.37	6.93	7.51	11.61	4.34	6.80	3.94	12.97	2.66	13.57	7.13	4.23	6.20	4.95	4.36	-			
18	30.19	24.67	14.94	10.43	15.93	5.29	18.36	3.94	12.97	2.66	13.57	7.13	4.23	6.20	0.36	0.73	6.37	-		
19	26.94	17.84	9.50	5.99	16.77	4.68	13.15	5.83	7.32	8.08	11.78	1.98	0.14	1.48	3.83	1.68	2.03	3.65	-	
20	46.04	37.26	26.60	25.57	25.07	15.97	27.80	13.89	23.50	9.15	26.52	17.77	13.14	18.29	6.33	8.49	13.31	7.39	12.10	-

Source: Authors (2023).

**Table 5:** Chemical characterization of the soil before the experiment was established. Bom Jesus, PI, UFPI, 2018.

pH	H+Al	Al	Ca	Mg	SB	T	P	K
H_2_O	-------------------cmolc dm^−3^--------------------						----mg dm^−3^------	
5.5	2.97	0.00	1.88	0.54	2.66	5.63	19.60	91.7

H+AL, potential acidity; Al, aluminum; Ca, calcium; Mg, magnesium; SB, sum of bases; T, CEC pH 7; P, phosphorus; K, potassium. Source: Authors (2023).

**Table 6:** Soil fertilization in the experimental area performed before, during, and after the experiment in Bom Jesus, PI, UFPI, 2018.

Fertilization Source *	Planting Value (g)	Fertilization Source	Topdressing Value (g)
(SFS)	300	Urea (20 DAE)	40
(KCl)	40	(NH_4_) 6Mo_7_O_24_ (25 DAE)	4.36
Urea	54	Urea (30 DAE)	40
(H_3_BO_3_)	3.53	Limestone (30 DAP)	450
(ZnSO_4_·7H_2_O)	9	-	-

* Chemical attributes: commercial product; Days after emergence, DAE; Days before planting, DAP. Source: Authors (2023).

## Data Availability

All the data is forming part of the work.
